# Case report: Free autologous costal cartilage transplantation for osteochondral lesions of the talus: three cases with 2–5 years follow-up

**DOI:** 10.3389/fbioe.2025.1556910

**Published:** 2025-03-05

**Authors:** Dajiang Du, Jiewei Chen, Che Zheng, Yun Gao, Mengxin Xue, Kaiwen Zheng, Peijun Xu, Jinyu Zhu, Changqing Zhang

**Affiliations:** ^1^ Department of Orthopedic Surgery, Shanghai Sixth People’s Hospital Affiliated to Shanghai Jiao Tong University School of Medicine, Shanghai, China; ^2^ Institute of Diagnostic and Interventional Radiology, Shanghai Sixth People’s Hospital Affiliated to Shanghai Jiao Tong University School of Medicine, Shanghai, China

**Keywords:** autografting, cartilage defect, costal cartilage, osteochondral lesion, talus

## Abstract

**Background:**

Osteochondral lesions of the talus (OLT) is a common and clinically challenging condition with no consensus on the optimal treatment. The prospective case series aim to evaluate the feasibility and clinical outcomes of free autologous costal cartilage transplantation (ACCT) for OLT.

**Methods:**

From April 2018 to October 2022, three patients who were diagnosed with OLT underwent free ACCT. Demographic characteristics, including age, gender, lesion size and location were collected at baseline. Functional and imaging outcomes were evaluated at 1 year, 2 years, and 5 years postoperatively. The primary outcomes were American Orthopedic Foot and Ankle Society (AOFAS) score and Foot and Ankle Ability Measure (FAAM) score. Secondary outcomes included Numeric Rating Scale (NRS), Tegner Activity Scale, and evaluations of images. A paired t-test was used for preoperative and postoperative comparison of the paired-design dataset.

**Results:**

Three patients (37.33 ± 16.50 years old) were included in the study with 2–5 years follow-up. AOFAS score improved from 60 ± 11 at baseline to 96 ± 6.93 at 2 years (p < 0.01) and 94 ± 8.49 at 5 years. FAAM/ADL improved from 60.97 ± 6.58 at baseline to 98 ± 1.83 at 2 years (p < 0.01) and 97 ± 0.85 at 5 years. FAAM/Sports improved from 56.4 ± 11.95 at baseline to 88.23 ± 11.34 at 2 years (p < 0.01) and 89 ± 4.67 at 5 years. Other functional scores in patient reported outcomes also showed significant improvements. Postoperative CT and MRI showed complete defect filling and robust tissue integration after ACCT. Arthroscopic evaluations further confirmed solid integration of costal cartilage into the underlying subchondral bone with a smooth surface over the repair site.

**Conclusion:**

Free ACCT is a feasible method for improving ankle function and quality of life for at least 2 years in patients with OLT. Promising long-term outcomes may be possible because of the good integration between the recipient talus and the implanted ACCT.

## Introduction

Up to 70% of sprains and fractures involving the ankle are thought to result in osteochondral lesions of the talus (OLT) (Mangwani et al., 2024). OLT represents a significant clinical challenge, as untreated OLT can lead to the development of osteoarthritis, which may result in chronic pain, functional impairment, and reduced quality of life (Hannon et al., 2014). The economic burden associated with OLT is substantial, encompassing both direct medical costs and indirect costs due to lost productivity.

OLT is seldom treated non-operatively as results of non-operative treatment have shown a success rate of less than 50% (Zengerink et al., 2010). Surgical treatment options for OLT can be broken down into bone marrow stimulation and tissue transplantation, and are frequently dictated by characteristics of the lesion (Mitchell et al., 2009). Bone marrow stimulation has been classically used for small defects, with optimal outcomes reported in lesions less than 1.5 cm^2^ in area, under 10 mm in diameter, and less than 5 mm in depth. Tissue transplantations, including osteochondral autograft transplantation (OAT) and autologous chondrocyte implantation (ACI), are used for larger defects (Erickson et al., 2018). However, these techniques are not without drawbacks. ACI requires two-stage surgery and a chondrocyte-loaded scaffold (Aurich et al., 2011; Kwak et al., 2014). The acquisition of osteochondral autograft can lead to significant trauma of the donor site, and bone tissue inside is difficult to cut and shape (Shi et al., 2022; Scranton et al., 2006). In light of these challenges, there is a pressing need for innovative approaches that can provide durable cartilage repair with minimal morbidity.

The ideal graft to repair OLT may possess highly similar structure and biological properties. Costal cartilage is the largest source of hyaline cartilage in the human body and is accessible without severe injury (Zhang et al., 2022). Free costal cartilage is easily moldable, facilitating to restore the smooth and even surface of weight-bearing joints. In the past decade, there have been more and more evidence proving the promising results of free autologous costal chondral transplantation for articular cartilage repair. Sato et al. (2018) reported favorable longer-term clinical results of autologous costal cartilage transplantation (ACCT) for advanced osteochondritis dissecans of the capitulum humeri in adolescent and young adult athletes. Zhang et al. (2022) reported a prospective study of autologous costal cartilage graft for osteochondral lesions of the femoral head. All patients achieved complete integration of grafts, and their function improved significantly for at least 3 years.

Despite the potential advantages of free ACCT, the application in OLT treatment has been underexplored. This study aimed to evaluate the clinical outcomes of free ACCT in OLT, with a focus on pain relief, functional recovery, and cartilage repair. We enrolled three patients from 2018, and reported the long-term outcomes of the first-time application of free ACCT in OLT.

## Methods

### Study design

The study was approved by Human Ethics Committee of Shanghai Sixth People’s Hospital Affiliated with the Shanghai Jiao Tong University School of Medicine (Approval No. 2018-027) and followed the Preferred Reporting Of Case Series in Surgery (PROCESS) guideline (Agha et al., 2020). Three patients were diagnosed with OLT based on clinical manifestations and MRI results. All of them underwent free ACCT and were enrolled in this study with at least 2 years follow-up.

The inclusion criteria were: age >18 years, sign of OLT under MRI, symptoms lasting for at least 3 months, and disabilities of daily life. Exclusion criteria included: rib fracture or deformity, active chest infection, a history of talar fracture, ankle arthritis with joint space narrowing, and ankle infection.

### Surgical process

**FIGURE 1:**
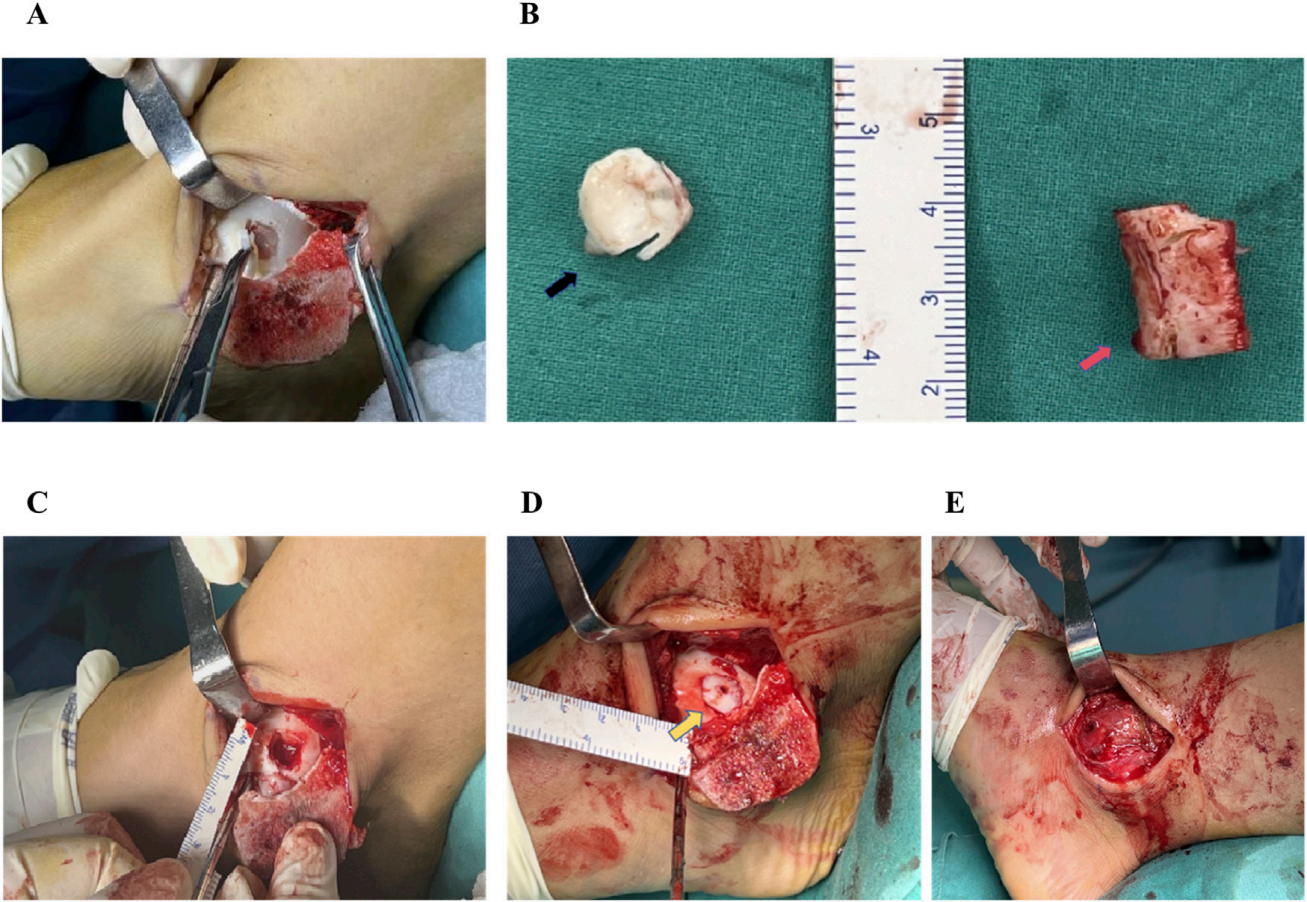
The surgical procedure of autologous costal cartilage transplantation for the treatment of talar cartilage lesion. **(A)** The exposure of the cartilage lesion to the medial aspect of the talar dome following malleolar Chevron osteotomy. **(B)**The damaged cartilage on the talar dome (black arrow) and a segment of costal cartilage approximately 2 cm in length (red arrow). **(C)** Using bone knives and bone curettes to debride the damaged cartilage, necrotic and devitalized tissue, as well as sclerotic bone, down to fresh bleeding bone bed. **(D)** Costal graft was transplanted into the cartilage defect site (yellow arrow) and fixed using an absorbable screw, combined additional trimming to match the congruency of the articular surface of the talus. **(E)** Following reduction of the medial malleolar fragment, two cannulated screws were inserted for stabilization.

Surgery was performed under general anesthesia with the patient in the supine position. A curved incision measuring approximately 10 cm in length was performed over the medial malleolus to achieve layered exposure. After the medial malleolar Chevron osteotomy, osteotomized medial malleolus was retracted inferiorly to achieve good exposure of the medial lesions of the talus (Figure 1A). Bone knives and bone curettes were used to debride the damaged cartilage, necrotic and devitalized tissue, as well as sclerotic bone, down to fresh bleeding bone bed (Figure 1C).

A 2-3 cm transverse incision was made above the sixth costal cartilage body on the right side. The attached muscle was dissected bluntly, and be careful to avoid damaging the pleura, intercostal nerve, and vessels. After complete exposure of the costal cartilage, a 2 cm long segment was harvested using the bone knives (Figure 1B). The segment of costal cartilage was trimmed according to the size and shape of the talus osteochondral defect to match the recipient site. After press-fitting into the lesion site, the graft was fixed using an absorbable screw (Freedom Screw; Inion). Additional trimming was performed to match the congruency of the articular surface of the talus (Figure 1D). Following reduction of the medial malleolar fragment, two cannulated screws were inserted for stabilization (Figure 1E).

Generally, the postoperative rehabilitation protocol was divided into three phases. Early phase (0–8 weeks): patients were instructed to maintain non-weight-bearing status to protect the surgical site. Gentle range-of-motion exercises for the ankle and subtalar joints were initiated to prevent stiffness while avoiding excessive stress on the reconstructed area. Intermediate phase (8–12 weeks): partial weight-bearing was allowed at 8 weeks postoperatively, using crutches or a walker for support. Progressive strengthening exercises for the lower extremity muscles were introduced, focusing on controlled loading and stability. Advanced Phase (>12 weeks): full weight-bearing was permitted at 12 weeks postoperatively, provided there was radiographic evidence of adequate healing. Patients were gradually transitioned to functional activities, including gait training and proprioceptive exercises, to restore normal mobility and strength.

### Patient-reported and imaging outcomes

Clinical and imaging evaluation were performed at baseline and postoperatively at 1 year, 2 years, and 5 years. The primary outcomes were the improvement in the American Orthopaedic Foot and Ankle Society (AOFAS) score (Imhoff et al., 2011), Foot and Ankle Activity Measure/Activities of Daily Living (FAAM/ADL) score, and Foot and Ankle Activity Measure/Sports (FAAM/Sports) score (Martin et al., 2005) from baseline. Secondary outcomes were other patient-reported outcomes including numeric rating score (NRS) (Breckenridge and McAuley, 2011) and Tegner score (Briggs et al., 2009), as well as imaging evaluations including International Cartilage Repair Society (ICRS) score (Brittberg and Winalski, 2003) and Magnetic Resonance Observation of Cartilage Repair Tissue (MOCART) score (Schreiner et al., 2024).

### Statistical analysis

Paired t-tests were used to assess functional and imaging outcomes (continuous variables). Statistical significance was set at a p-value of less than 0.05. All analyses were performed in R studio (version 4.1.3)

## Results

### Baseline characteristics

The baseline characteristics of the three patients are presented in Table 1. The mean age of three patients was 37.33 ± 16.50 years. The average area of the cartilage lesions was 105 ± 18 mm^2^, with an average lesion depth of 4.7 ± 0.6 mm.

**TABLE 1:** Baseline characteristics of included patients.

Patient no.	Gender	Age	Follow-up duration (years)	Lesion site	Lesion area (cm^2)	Depth (cm)
①	Male	18	2	Right (Medial)	1.1*0.9	0.4
②	Male	50	6	Left (Medial)	1.4*0.9	0.5
③	Female	44	5	Left (Medial)	1.5*0.6	0.5

### Clinical function

**FIGURE 2:**
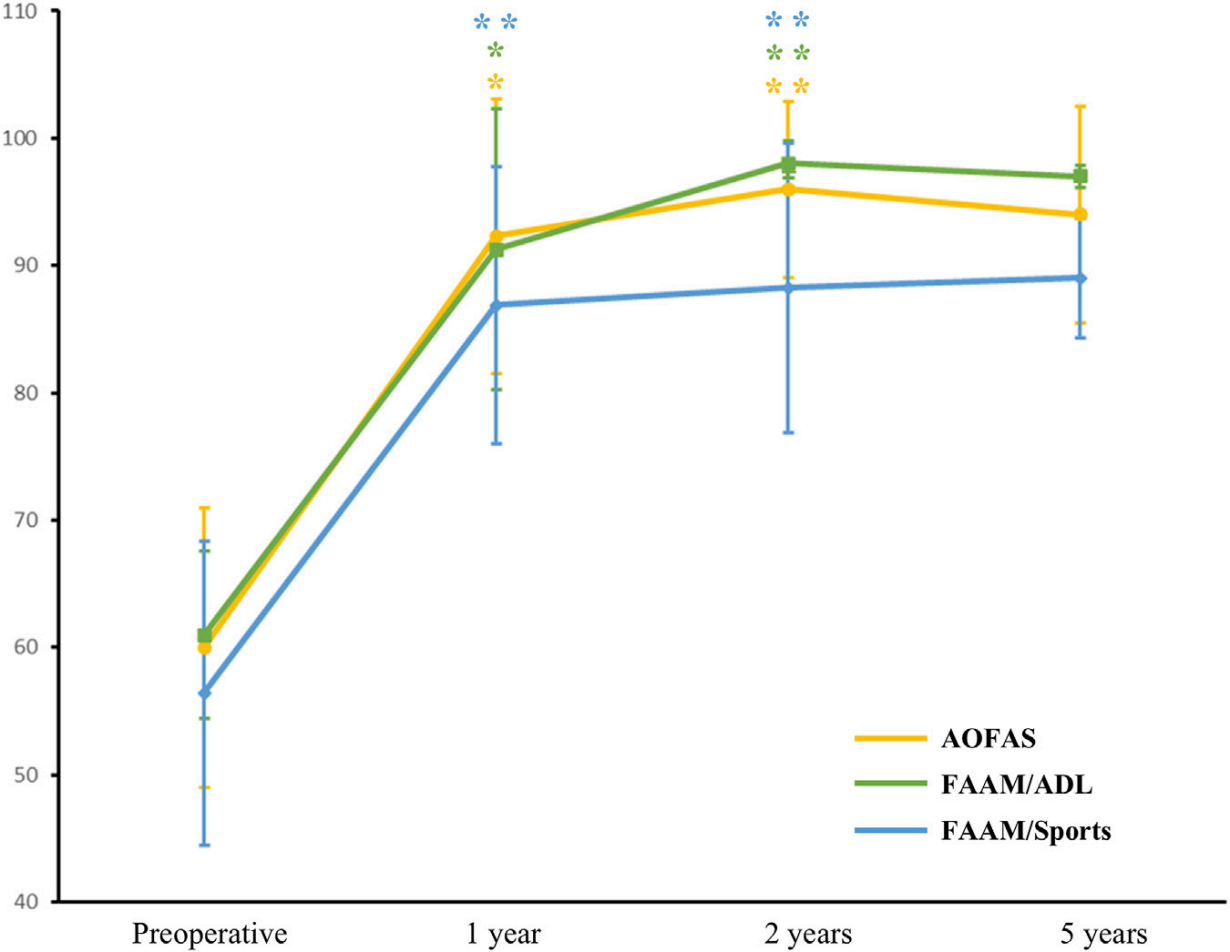
Evaluations of functional outcomes at baseline and after surgery. Results were presented as mean (points on line) and 95%CI (error bars). Level of significance: *, p < 0.05; **, p < 0.01. *Abbreviations*: AOFAS, American Orthopaedic Foot and Ankle Society score; FAAM/ADL, Foot and Ankle Activity Measure/Activities of Daily Living score; FAAM/Sports, Foot and Ankle Activity Measure/Sports score.

Over a long-term follow-up, no patients reported pain, and all functional outcomes reached expected levels. At 2 years after surgery, AOFAS (from 60 ± 11 to 96 ± 6.93, p < 0.01), FAAM/ADL (from 60.97 ± 6.58 to 98 ± 1.83, p < 0.01), and FAAM/Sports (from 56.4 ± 11.95 to 88.23 ± 11.34, p < 0.01) showed significant improvement from baseline (Figure 2). Similarly, NRS decreased from 5 ± 1 to 1.33 ± 0.58 (p = 0.03), and Tegner score increased from 3.67 ± 0.58 to 6.33 ± 1.15 (p = 0.01). At 5 years after surgery, AOFAS score improved from 60.00 ± 15.56 at baseline to 94 ± 8.49, FAAM/ADL score improved from 61.35 ± 9.26 at baseline to 97.00 ± 0.85, and FAAM/ADL score improved from 51.80 ± 12.59 at baseline to 89.00 ± 4.67 (Figure 2). Similar findings could be seen in NRS (from 5.50 ± 0.71 to 1.50 ± 0.71) and Tegner score (from 3.50 ± 0.71 to 6.00 ± 1.41). Postoperative ankle function was nearly restored to normal. Patients were able to walk, run, jump, squat, and balance on one leg without pain or significant restriction (Supplementary Figure S1).

### Imaging evaluations

**FIGURE 3:**
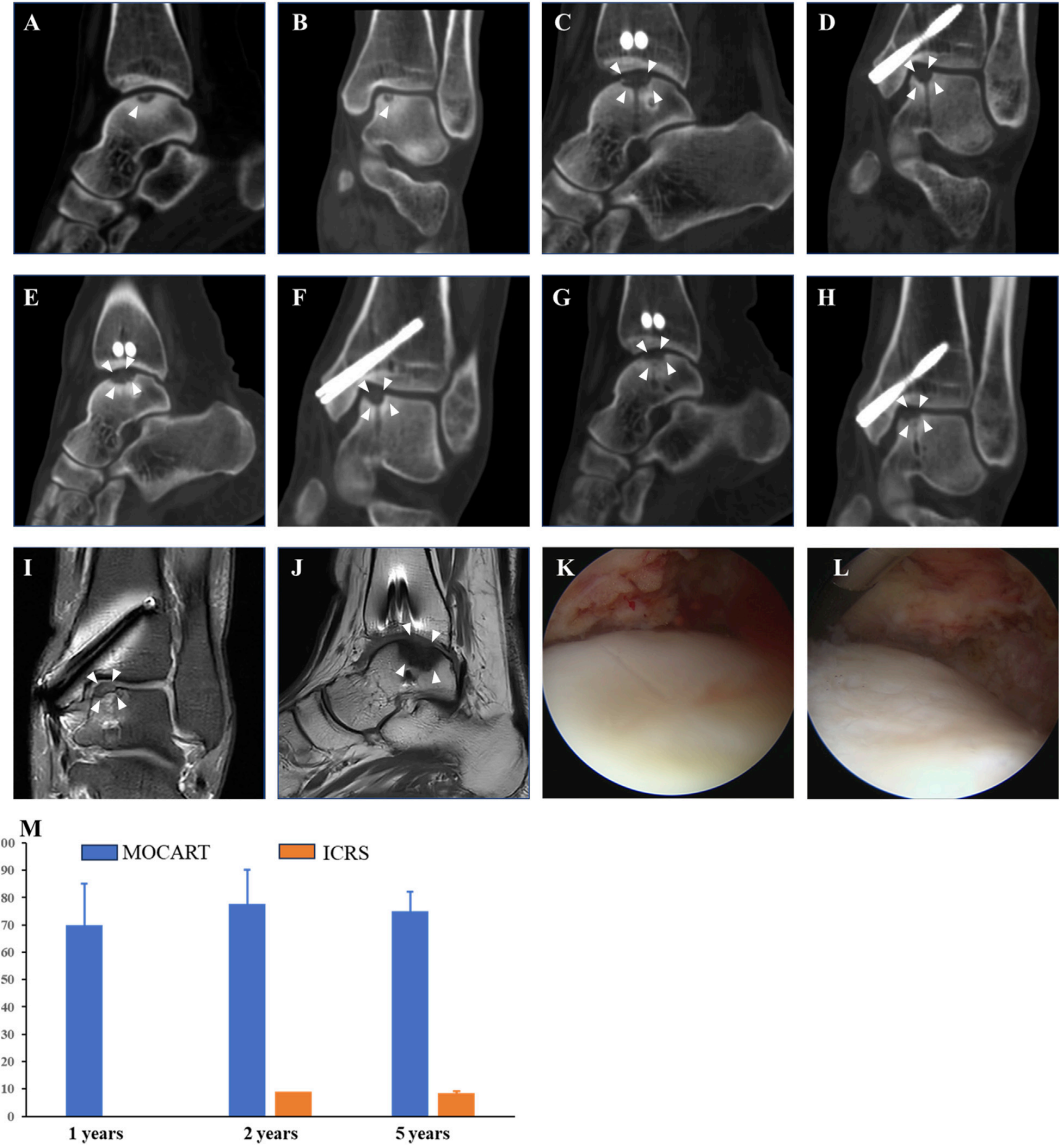
Evaluations of Imaging outcomes. 44-year-old women (patient NO.3) who were diagnosed with OLT and underwent free ACCT in 2018 with a follow-up for 5 years. The MOCART score was 70 at 1 year, 78 at 2 years, and 77 at 5 years. The ICRS score was 9 under second-look arthroscopy. **(A, B)** Preoperative coronal and sagittal planes of CT. **(C, D)** Coronal and sagittal planes of CT at 1 year after surgery. **(E, F)** Coronal and sagittal planes of CT at 2 years after surgery. **(G, H)** Coronal and sagittal planes of CT at 5 years after surgery. **(I, J)** Coronal and sagittal planes of MRI at 5 years after surgery. **(K, L)** Arthroscopy at 5 years after surgery. **(M)** MOCART and ICRS scores. White arrow, region of interest. *Abbreviations*: ICRS, International Cartilage Repair Society score; MOCART, Magnetic Resonance Observation of Cartilage Repair Tissue score.

Compared with preoperative imaging (Figures 3A, B), postoperative assessments (Figures 3C–J) at 1 year, 2 years, and 5 years demonstrated complete defect filling, robust tissue integration, and signals were nearly identical with native cartilage. The arthroscopy revealed a smooth, continuous surface over the repair site, with tissue appearing firm and stable. The repaired surface was continuous with adjacent cartilage and shows solid integration into the underlying subchondral bone (Figures 3K, L). MOCART score based on MRI was 77.67 ± 12.5 at 2 years and 75 ± 7.07 at 5 years. ICRS score based on second-look arthroscopy was 9 at 2 years and 8.5 ± 0.71 at 5 years (Figure 3M).

## Discussion

To our knowledge, this was the first application of free ACCT in treating OLT (back to 2018) and first report about the long-term clinical results of this technique. In this study, we employed a case series design to evaluate the clinical efficacy of ACCT in treating OLT, with a focus on long-term functional and radiographic outcomes. Our findings demonstrated significant improvements in patient-reported outcomes, including pain relief and functional recovery, as well as robust tissue integration observed through advanced imaging techniques. These results suggested that ACCT might offer a viable alternative for OLT management, particularly in cases where traditional treatments have failed. The following discussion will delve into the implications of these findings, compare ACCT with existing therapies, and explore the factors influencing treatment success, while also addressing the limitations of our study and proposing directions for future research.

### Bone marrow stimulation (BMS)

BMS encompasses a range of techniques aimed at activating the subchondral bone marrow to facilitate cartilage repair. BMS technique is primarily suitable for OLT patients with small defects (area <1.5 cm^2^, diameter <10 mm, and depth <5 mm) (Powers et al., 2021). Compared with free ACCT, BMS showed limited value of application for OLT with large lesions, as two patients had lesion depths exceeding 5 mm, and all patients had a lesion diameter greater than 10 mm. In addition, the resulting fibrocartilage after BMS typically lacks the mechanical durability of native hyaline cartilage, with its biological and biomechanical properties being markedly inferior. Over time, fibrocartilage tends to degenerate, leading to lower survival rates and less favorable clinical outcomes in long-term follow-up (O'Loughlin et al., 2010). This stands in stark contrast to the complete defect filling and robust tissue integration after ACCT for 5 years.

However, the proximity of patients’ lesion size to BMS criteria makes it challenging to definitively demonstrate the superiority of free ACCT over BMS in this study. A prospective comparative study incorporating a BMS control group is warranted in future research. To better demonstrate the potential advantages of free ACCT, particularly for larger lesions, we introduced another 31-year-old woman (NO. 4) who was diagnosed with giant cell tumor of tendon sheath (GCTTS) of talus. The area of lesion was 1.6*1.5 cm^2^, and the depth of lesion was 1.3 cm. This patient underwent free ACCT with a follow-up for 1 year. At 1 year after surgery, functional scores and imaging outcomes of this patient showed significant improvements. The surgical procedure, patient reported outcomes, and imaging evaluations of this patient were summarized in Supplementary Material. Although this case was diagnosed with GCTTS rather than OLT, it effectively demonstrated superiority of free ACCT in treating larger defects and versatility of the technique across different pathologies.

### Autologous chondrocyte implantation (ACI)

ACI is applied in two stages, the first stage involves extraction of articular cartilage tissue and cell cultivation, and the second stage involves transplantation surgery after three to 4 weeks of cell cultivation. In a study by Kwak et al. (2014), 29 OLT patients after ACI for 2–10 years demonstrated sustained pain relief and satisfactory clinical outcomes (Supplementary Table S1). Giannini et al. (2014) applied ACI in the treatment of OLT and observed a significant clinical improvement with the AOFAS score increasing from 57.2 preoperatively to 89.5 at 3 years follow-up (Supplementary Table S1). Compared with free ACCT, ACI comprises two surgical procedures and one cell cultivation process, which will obviously increase the hospital stay and economic burden of patients. Besides, autologous chondrocytes lack initial stability and require a chondrocyte-loaded scaffold, including but not limited to periosteal (Baums et al., 2006), hyaluronan (Giannini et al., 2014), and collagen scaffold (Anders et al., 2012) (Supplementary Table S1). Thus, ACCT was convenient to implement and operate without additional require of scaffolds comparing with ACI.

### Autologous osteoperiosteal transplantation (AOPT)

Autologous osteoperiosteal is usually collected from iliac or distal tibia (Supplementary Table S1). Guo et al. (2023) applied AOPT in the treatment of OLT and observed a significant clinical improvement with the AOFAS score increasing from 69.2 preoperatively to 80.9 at 2 years follow-up (Supplementary Table S1). Similarly, Cao et al. (2024) enrolled 31 patients with 2.5 years follow-up and found that AOPT for OLT demonstrated good clinical efficacy with a low incidence of complications (Supplementary Table S1). Two retrospective cohort studies (Shi et al., 2022; Yang et al., 2024) have compared APOT with OAI or BMS, and APOT showed comparable clinical outcomes with OAI and better outcomes than BMS. However, in the first study (Guo et al., 2023), the degree of improvement in the overall pain and function scores after 2.5 years was relatively limited, with several individuals’ pain and function levels remaining unchanged, and a few even reported slightly worsened pain and function. In addition, second-look arthroscopy showed fibrillated tissue coverage in most AOPT cases (82%), and the surface of the fibrillated tissue was slightly higher than the surrounding normal cartilage. Comparing with AOPT, ACCT showed significant and stable improvements in long-term functional and radiographic outcomes.

### Osteochondral autograft transplantation (OAT)

OAT transfer typically entails harvesting one or more osteochondral cylindrical grafts, comprising both articular cartilage and subchondral bone. Gautier et al. (2002) and Scranton et al. (2006) collected osteochondral autograft from knees and found that OAT could improve clinical functions of OLT patients without adverse effects on the knee (Supplementary Table S1). Suh et al. enrolled 11 OLT patient and treated then with OAT from the lateral talar articular facet (Suh et al., 2024). This study found that AOFAS score increased from 55.4 preoperatively to 92.1 at 1 year follow-up. However, osteochondral autografts are usually collected from non-weight-bearing sites of weight-bearing joints such as knees and ankles, and long-term effects on the donor site require further investigation. In addition, subchondral bone in osteochondral autografts makes them hard to cut and shape, and not to mention that their sources are extremely limited. Costal cartilage is the main source of hyaline cartilage in the human body and is easily shaped. Comparing with OAT, ACCT had few influences on weight-bearing joints such as knees.

### Autologous costal cartilage transplantation (ACCT)

Costa consists of hyaline cartilage and bone, and its cartilage may be a feasible and effective graft for OLT. Previous studies have proved the promising and satisfactory outcomes of costal chondral/osteochondral transplantation in fingers, wrist, elbow and knee (Zhang et al., 2022; Sato et al., 2018; Shimada et al., 2012). Wei et al. (2023) reported using osteochondral autograft from costa as a treatment for OLT (Supplementary Table S1). Five patients with 1 year follow-up showed significant improvements in clinical and imaging outcomes. However, the graft was collected from osteochondral junction, which is close to the pleura, making it easy to damage the pleura during sampling. In addition, the bone tissue in the graft made it hard to cut and shape. The cross-sectional area of osteochondral autograft from costa was limited, resulting in a restricted repair area, and it might even require the use of two ribs, leading to significant damage. Thus, we considered using free ACCT to avoid the aforementioned shortcomings. Zhang et al. (2022) reported a prospective study of ACCT for osteochondral lesions of the femoral head. All patients achieved complete integration of grafts, and their function improved significantly for at least 3 years, which indicated possible application of ACCT in weight-bearing joints such as ankles.

### Strengths and limitations

The clinical implications of our findings were substantial, as they suggested that costal cartilage transplantation could become a standard treatment option for OLT, particularly in cases where other methods have proven ineffective. The significant improvements of clinical function had the potential to influence clinical guidelines, encouraging the adoption of ACCT as a first-line surgical intervention for OLT. Moreover, the robust tissue integration and near-native cartilage signals observed in imaging evaluations underscored the procedure’s durability, which could reduce the need for revision surgeries and lower healthcare costs associated with long-term management of OLT. While the current study employed traditional open surgical techniques, we recognize the potential of minimally invasive surgery and computation-based precise design in the future management of OLT. Minimally invasive approaches, such as arthroscopy-assisted ACCT, could reduce surgical trauma, shorten recovery times, and lower the risk of postoperative complications. Additionally, computation-based precise design, including 3D printing and computer-assisted surgical planning, could optimize the shape and size of cartilage grafts, ensuring better anatomical fit and integration. These technologies may also facilitate personalized treatment strategies, tailoring the procedure to the patient’s specific anatomical and pathological characteristics. Future studies should explore the feasibility and efficacy of these advanced techniques to further refine the surgical management of OLT.

This study had several limitations that warranted consideration. First, the small sample size of three patients limited the generalizability of the findings. While the results were promising, they might not fully represent the broader population of patients with OLT. Additionally, the lack of a control group or comparative treatment arm restricted the ability to draw definitive conclusions about the superiority of ACCT over other surgical techniques. Long-term follow-up, though comprehensive, was conducted in a single center, which might introduce bias. Furthermore, the study relied heavily on subjective patient-reported outcomes and imaging assessments, which, while valuable, might not capture all nuances of cartilage repair and functional recovery. Finally, although current study relied primarily on imaging evidence (CT, MRI, and endoscopy) to assess tissue integration and defect filling, we recognize the importance of histological evaluations in confirming the therapeutic outcome, which could provide direct evidence of cartilage and subchondral bone regeneration. However, due to ethical and practical constraints, we were unable to perform histological assessments in this study. Future research should aim to address these limitations through larger, multicenter, randomized controlled trials with standardized outcome measures.

## Conclusion

This study demonstrated that ACCT was a viable and effective treatment option for OLT, as evidenced by significant improvements in clinical function and robust imaging outcomes over a 5-year follow-up period. The findings suggested that ACCT could restore near-normal ankle function and provided durable cartilage repair. However, the study’s limitations, including its small sample size and lack of a control group, highlighted the need for further investigation. Future studies should focus on larger cohorts, comparative effectiveness analyses, and long-term outcomes to validate these results and refine the surgical approach. Despite these limitations, this study contributed valuable insights into the potential of ACCT as a promising therapeutic strategy for OLT.

## Data Availability

The original contributions presented in the study are included in the article/Supplementary Material, further inquiries can be directed to the corresponding authors.
