# Supplementary material for: Case report: Free autologous costal cartilage transplantation for osteochondral lesions of the talus: three cases with 2–5 years follow-up
Source: Front Bioeng Biotechnol. 2025 Mar 5;13:1556910. doi: 10.3389/fbioe.2025.1556910 (PMC11920135; doi:10.3389/fbioe.2025.1556910)
Supplement: Supplementary file 1 [file DataSheet1.docx]

**Supplementary Material**


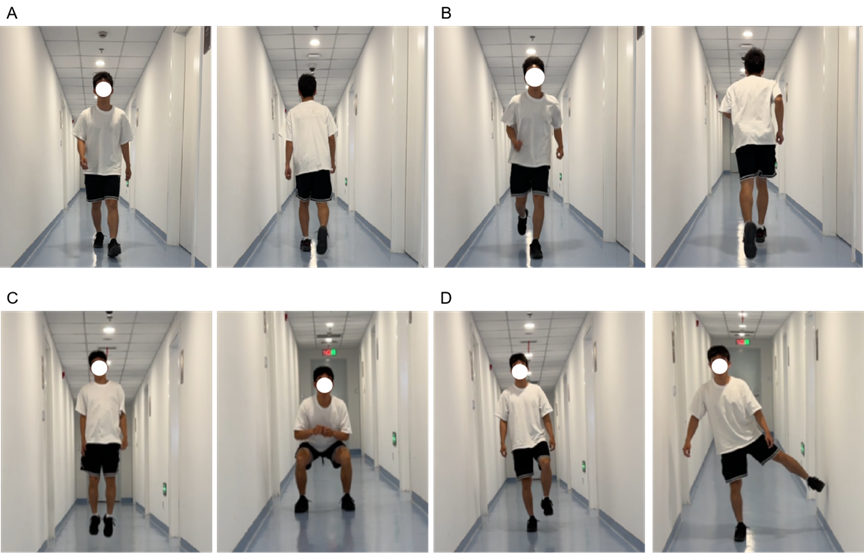


**Figure S1.** Case presentation of an 18-year-old patient: Right ankle function at 24 months postoperatively. (A) Walking test in a weight-bearing state. (B) Running test in a weight-bearing state. (C) Jumping and squatting test. (D) Single-leg standing test.

**Table S1. A literature review of current options of autologous graft for treating OLT.**

| **Author and year** | **Participants** | **Study type** | **Follow-up** | **Graft** | **Characteristics of lesions** | **Outcomes** |
| --- | --- | --- | --- | --- | --- | --- |
| Kwak 2014 ^[7]^ | 29 | Case series | 2-10 years | Autologous chondrocyte from knee, combined periosteal graft from the proximal or distal tibia | 1.98 cm^2^ in area | AOFAS, Tegner, Finsen, Imaging evaluations |
| Giannini 2014 ^[21]^ | 46 | Case series | 1.2 years | Autologous chondrocyte from knee, combined hyaluronan scaffold | 1.6 cm^2^ in area | AOFAS, Histological analysis |
| Baums 2006 ^[22]^ | 12 | Case series | 5 years | Autologous chondrocyte from anterior part of the talus, combined a periosteal flap from the distal tibia | 2.3 cm2 in area | AOFAS, Hannover ankle score |
| Anders 2012 ^[23]^ | 22 | Case series | 5 years | Autologous chondrocyte from lesion site of the talus, combined a collagen type I/III scaffold | 1.94 cm2 in area | AOFAS, VAS, Tegner, MOCART |
| Gautier 2002 ^[27]^ | 11 | Case series | 2 years | Osteochondral autograft from non-weight-bearing trochlear border of the ipsilateral knee | 18 mm*10mm in area | AOFAS, Hannover ankle score |
| Scranton 2006 ^[9]^ | 50 | Case series | 3 years | Osteochondral autograft from knee | 8 mm to 20 mm in diameter | Karlsson-Peterson Ankle Score |
| Wei 2023 ^[30]^ | 5 | Case series | 1 year | Osteochondral autograft from ribs | 10.40±1.86mm in depth; 10.66±1.84mm in diameter | AOFAS, FAAM, MOCART, ICRS |
| Suh 2024 ^[28]^ | 11 | Case series | 5 years | Osteochondral autograft from the lateral talar articular facet | 7.3 mm in depth;  8 to 10 mm in diameter | AOFAS, VAS, MOCART |
| Shi 2022 ^[8]^ | 46 | Retrospective cohort study | 4 years | Osteochondral autograft from knee vs Autologous osteoperiosteal from iliac | 2.17 cm^3^ or 2.55 cm^3^ in volume | AOFAS, VAS, Tegner, MOCART, ICRS |
| Cao 2024 ^[25]^ | 31 | Case series | 2.5 years | Autologous osteoperiosteal and cancellous bone from iliac or distal tibia | 8.5±1.8mm in depth; 81.1±37.4mm^2^ in area | AOFAS, VAS, SF‐36 |
| Guo 2022 ^[24]^ | 26 | Case series | 2.5 years | Autologous osteoperiosteal from iliac, combined recombinant human bone morphogenetic protein 2 | 10.7±3.5mm in depth;  13.6±4.0mm in diameter | FFI, AOFAS, VAS, Tegner, MOCART |
| Yang 2024 ^[26]^ | 55 | Retrospective cohort study | 5 years | Autologous osteoperiosteal from iliac vs bone marrow stimulation | AOPT group: 11.7 ± 2.8 mm in depth; 126.5±18mm2 in area | AOFAS, VAS, FAAM |

*Abbreviations*: AOFAS, American Orthopaedic Foot and Ankle Society score; FAAM, Foot and Ankle Activity Measure; ICRS, International Cartilage Repair Society score; MOCART, Magnetic Resonance Observation of Cartilage Repair Tissue score; VAS, Visual Analogue Scale; SF‐36, 36 item Short‐Form Health Survey; FFI, Foot Function Index; OLT, osteochondral lesions of the talus; AOPT, autologous osteoperiosteal transplantation.

**Table S2. Baseline characteristics of NO.4 patient.**

| Patient No. | Gender | Age | Follow-up Duration (years) | Lesion site | Lesion area (cm^2) | Depth (cm) |
| --- | --- | --- | --- | --- | --- | --- |
| ④ | Male | 31 | 1 | Left (Medial) | 1.6*1.5 | 1.3 |

**Table S3.** **Functional and Radiographic Outcomes of NO.4 patient**

| Patient No. | AOFAS | |  | NRS | |  | FAAM/ADL | |  | FAAM/Sports | |  | Tegner | |
| --- | --- | --- | --- | --- | --- | --- | --- | --- | --- | --- | --- | --- | --- | --- |
|  | Pre | 1 year |  | Pre | 1 year |  | Pre | 1 year |  | Pre | 1 year |  | Pre | 1 year |
| 4 | 50 | 85 |  | 5 | 2 |  | 54.8 | 96.4 |  | 42.9 | 75 |  | 4 | 7 |

AOFAS, American Orthopaedic Foot and Ankle Society score; FAAM/ADL, Foot and Ankle Activity Measure/Activities of Daily Living score; FAAM/ Sports, Foot and Ankle Activity Measure/Sports score.


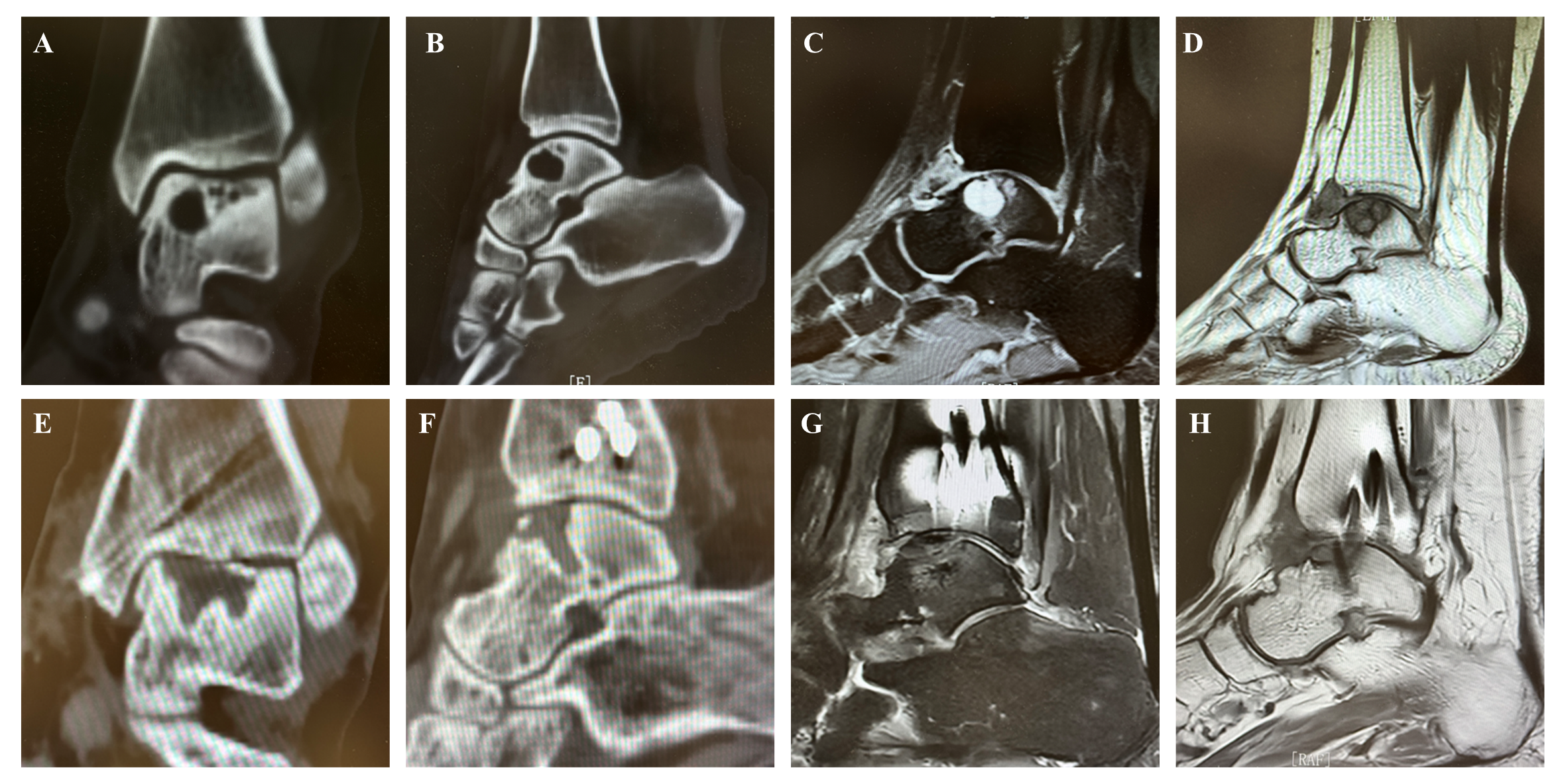


**Figure S2. Evaluations of preoperative and 1-year postoperative imaging outcomes.**


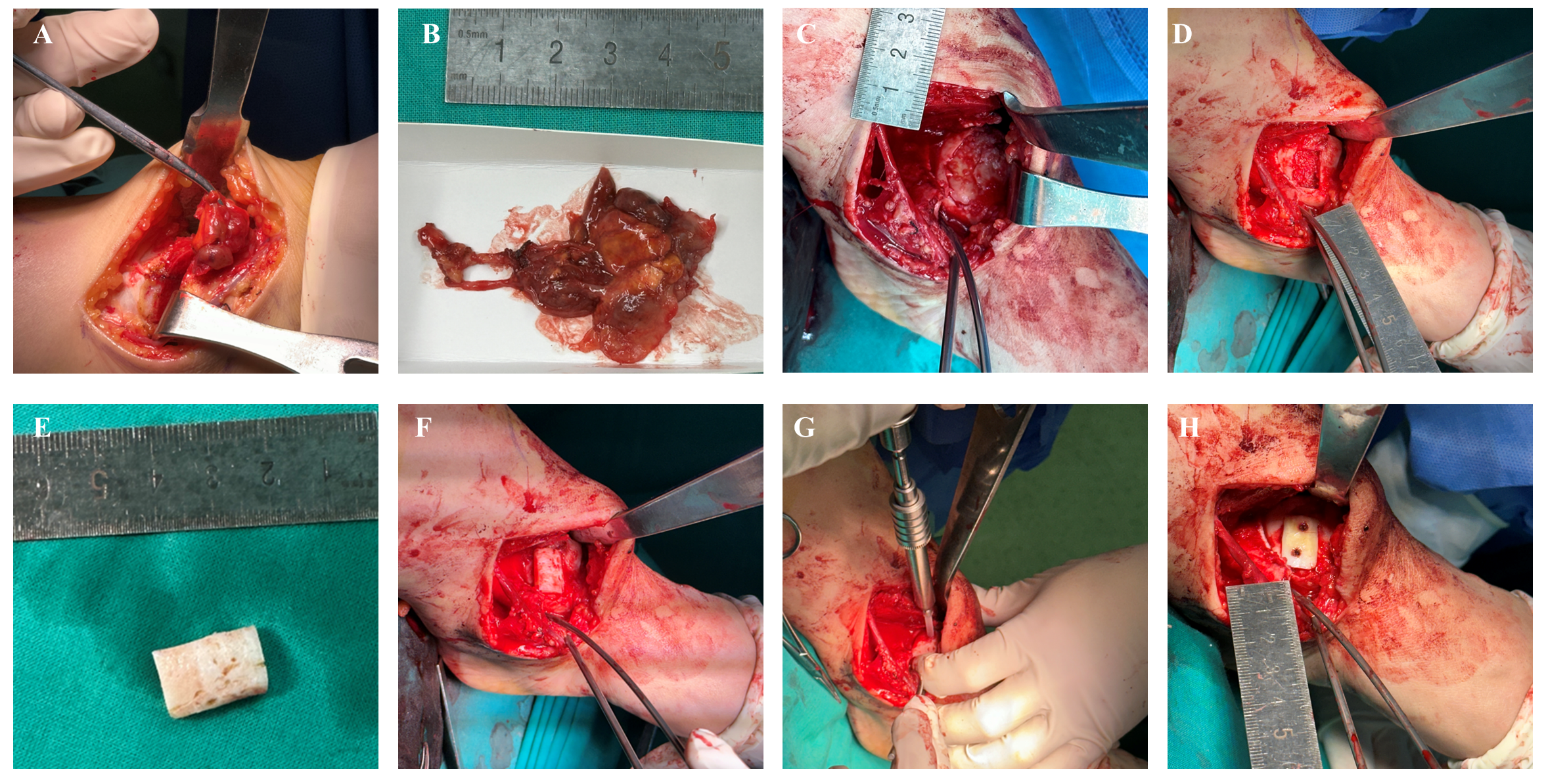


**Figure S3. The surgical procedure of autologous costal cartilage transplantation for the treatment of giant cell tumor of tendon sheath (GCTTS) of talus.**


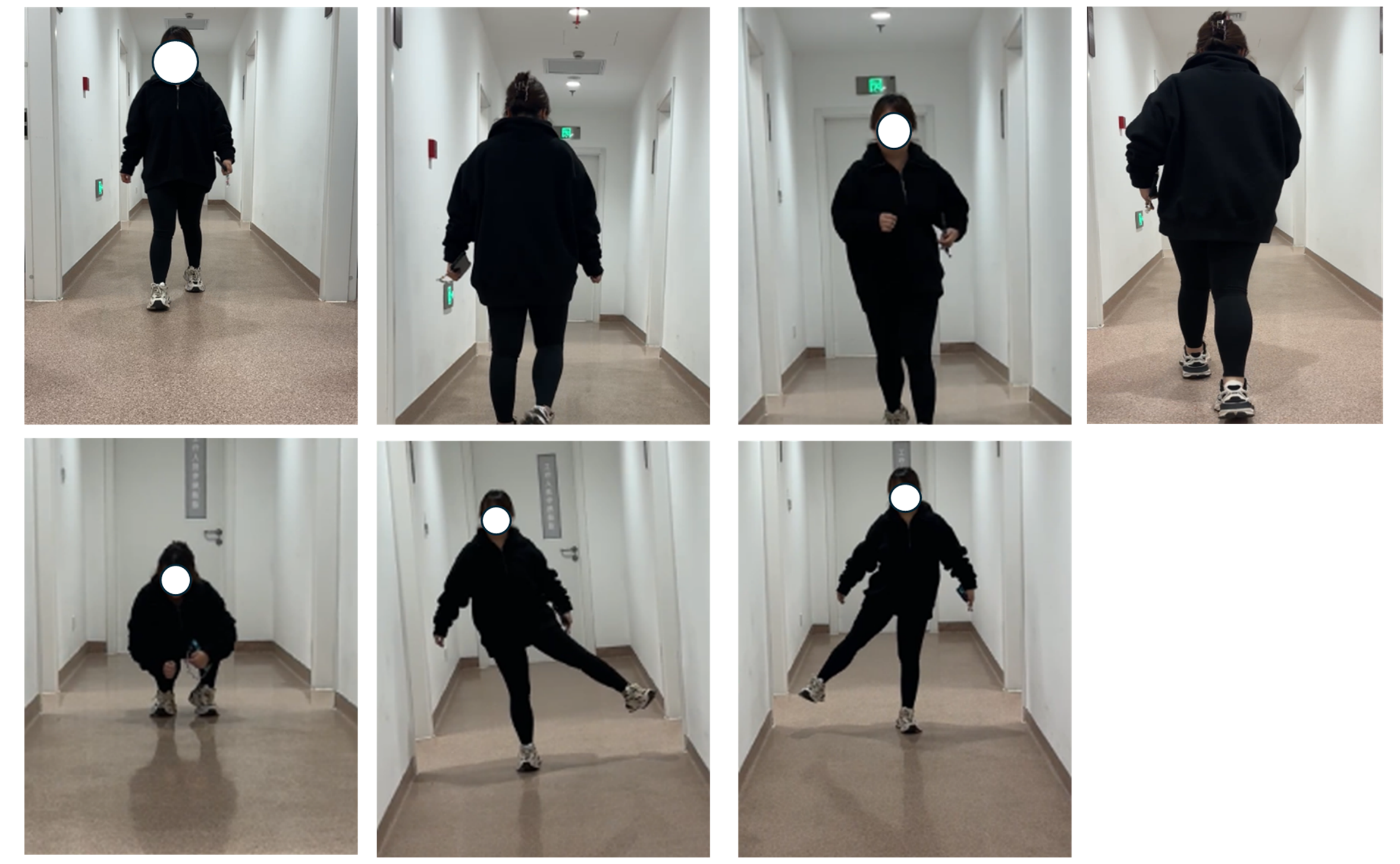


**Figure S4. Postoperative ankle function of NO.4 patient.**
